# The antibacterial activity of a prophage-encoded fitness factor is neutralized by two cognate immunity proteins

**DOI:** 10.1016/j.jbc.2024.108007

**Published:** 2024-11-16

**Authors:** Andrea G. Alexei, Nathan P. Bullen, Stephen R. Garrett, David Sychantha, John C. Whitney

**Affiliations:** 1 Michael DeGroote Institute for Infectious Disease Research, McMaster University, Hamilton, Ontario, Canada; 2 Department of Biochemistry and Biomedical Sciences, McMaster University, Hamilton, Ontario, Canada

**Keywords:** ADP-ribosylation, bacterial toxin, bacteriophage, polymorphic toxins, ADP-ribosyl hydrolase

## Abstract

The human gastrointestinal tract is a competitive environment inhabited by dense polymicrobial communities. *Bacteroides*, a genus of Gram-negative anaerobes, are prominent members of this ecological niche. *Bacteroides* spp. uses a repertoire of mechanisms to compete for resources within this environment such as the delivery of proteinaceous toxins into neighbouring competitor bacteria and the ability to consume unique metabolites available in the gut. In recent work, *Bacteroides stercoris* gut colonization was linked to the activity of a prophage-encoded ADP-ribosyltransferase, which was found to stimulate the release of the metabolite inosine from host epithelial cells. This fitness factor, termed Bxa, shares a similar genomic arrangement to bacterial toxins encoded within interbacterial antagonism loci. Here, we report that Bxa also possesses antibacterial ADP-ribosyltransferase activity, raising the question of how Bxa-producing bacteria resist intoxication prior to Bxa’s release from cells. To this end, we identify two cognate immunity proteins, Bsi and BAH, that neutralize Bxa’s antibacterial activity using distinct mechanisms. BAH acts as an enzymatic immunity protein that reverses Bxa ADP-ribosylation whereas Bsi physically interacts with Bxa and blocks its ADP-ribosylation activity. We also find that the N-terminal domain of Bxa is dispensable for toxicity and homologous domains in other bacteria are fused to a diverse array of predicted toxins found throughout the Bacteroidaceae, suggesting that Bxa belongs to a broader prophage encoded polymorphic toxin system. Overall, this work shows that Bxa is a promiscuous ADP-ribosyltransferase and that *B. stercoris* possesses mechanisms to protect itself from the toxic activity of this prophage encoded fitness factor.

The human gastrointestinal tract is home to a dense polymicrobial community whose members comprise microbes from all domains of life (1, 2, 3). However, in this ecological niche, the microbiota of healthy adults is primarily dominated by two bacterial phyla: the *Bacillota* and *Bacteroidetes* (4). One of the most abundant commensals throughout the gastrointestinal tract are the obligately anaerobic *Bacteroides*, a genus belonging to the *Bacteroidetes* phylum (5). Given their prominence, there has been a concerted effort to understand what underlying molecular mechanisms facilitate *Bacteroides*’ successful colonization of such a competitive and ecologically dense environment.

The stable communities formed by *Bacteroides* within the gut are, in part, attributed to their ability to directly antagonize other bacteria. For example, *Bacteroides fragilis*, a common member of the mammalian gut microbiome, can outcompete neighboring bacteria through the delivery of polymorphic proteinaceous toxic effectors into adjacent cells *via* the type VI secretion system (T6SS) (6, 7). The diverse effectors of the T6SS, which include cytoplasmic and cell-surface acting toxins, often contain conserved N-terminal trafficking domains that target them to the T6SS apparatus (8). To protect from self-intoxication prior to effector export and from intercellular effector delivery, toxin producing cells express immunity proteins that neutralize toxin activity (8). Analysis of sequenced *Bacteroides* gut microbiome residents reveals that at least half of the strains present in the gut encode a T6SS, indicating that T6SS-mediated killing is a widespread strategy for bacterial competition by this genus (9). The interbacterial antagonism mediated by the T6SS is thought to not only enable *Bacteroides* persistence in the gut microbiome but also play a role in shaping the microbial composition of this environment (10).

In addition to directly antagonizing competitor bacteria, *Bacteroides*’ abundance can also be attributed to their specialized metabolism, which uses unique host- and diet-derived metabolites unavailable to other members of the gut microbiome (5). To achieve this, many *Bacteroides* encode polysaccharide utilization loci (PUL), gene clusters containing enzymes dedicated to the degradation and transport of complex glycans (11). For instance, three of *Bacteroides thetaiotaomicron*’s 88 total PULs have been shown to mediate the breakdown of yeast-derived α-mannose, as well as the subsequent sequestration of the resulting simpler oligosaccharides. This latter process specifically excludes other microbiome constituents from utilizing the resulting sugars as energy sources (12, 13). Additionally, *B. thetaiotaomicron* encodes ribose-utilization systems (RUS), which are gene clusters that allow for nucleotides and nucleosides to be used as carbon sources (14). The catabolism of digestion-resistant glycans and other unconventional metabolites is a strategy unique to *Bacteroides*, suggesting that this genus persists in the gut through the formation of distinct nutritional niches. The broad metabolism of *Bacteroides* and the direct elimination of neighboring bacteria by T6SS-mediated antagonism represent only a subset of the mechanisms that contribute to *Bacteroides’* competitive fitness within the gut microbiome.

In recent work, Brown *et al.* identified a novel ADP-ribosyltransferase, Bxa, encoded by the ΦBxa prophage of *Bacteroides stercoris* that promotes gut colonization (15). Bxa was shown to ADP-ribosylate non-muscle myosin II within host epithelial cells and stimulate inosine release by the epithelia (15). Given that *B. stercoris* can use inosine as a carbon source, it is hypothesized that catabolism of this nucleoside contributes to successful gut colonization. In support of this, *B. stercoris* lysogens lacking *bxa* are unable to colonize the murine gastrointestinal tract to the level of their wild-type counterparts (15). While prophage-encoded gene products have been found to increase the fitness of the bacterial host, Bxa is unique among these in that is not a stand-alone enzyme but rather represents a C-terminal fusion to a minor capsid component, MuF, encoded in the head morphogenesis region of the bacteriophage ΦBxa (15, 16, 17, 18). Notably, the MuF domain has been previously identified in prophage-encoded antibacterial toxins and in this context is hypothesized to function as a capsid targeting domain (8, 19). While the biological role of MuF containing toxins is unknown, they share a similar toxin-immunity gene arrangement observed in bacterial toxin export systems such as the T6SS (8, 19). To date, the only characterized members of this family are the Apk2 proteins, which inhibit bacterial growth by catalyzing the production of the toxic alarmone (p)ppApp (20, 21).

Motivated by Bxa’s similarity to MuF toxins, we investigated whether it also possesses antibacterial activity. To this end, we found that Bxa is a potent antibacterial toxin that promiscuously ADP-ribosylates arginine residues on numerous protein targets. Furthermore, we discovered that two small open reading frames located adjacent to *bxa* encode cognate immunity proteins. One of these immunity proteins functions as an ADP-ribosyl hydrolase belonging to the ARH1 family whereas the other blocks Bxa activity through an inhibitory protein-protein interaction. Based on these findings, we also investigated whether homologs of Bxa are associated with other toxin domains and identified genes predicted to encode glutamine deaminases and alarmone synthetase toxins. Altogether, our findings reveal that Bxa is a member of the MuF bacterial toxin family and that its homologs are identified broadly throughout the Bacteroidaceae.

## Results

### Bxa ADP-ribosyltransferase activity causes bacterial cell death

**Figure 1:**
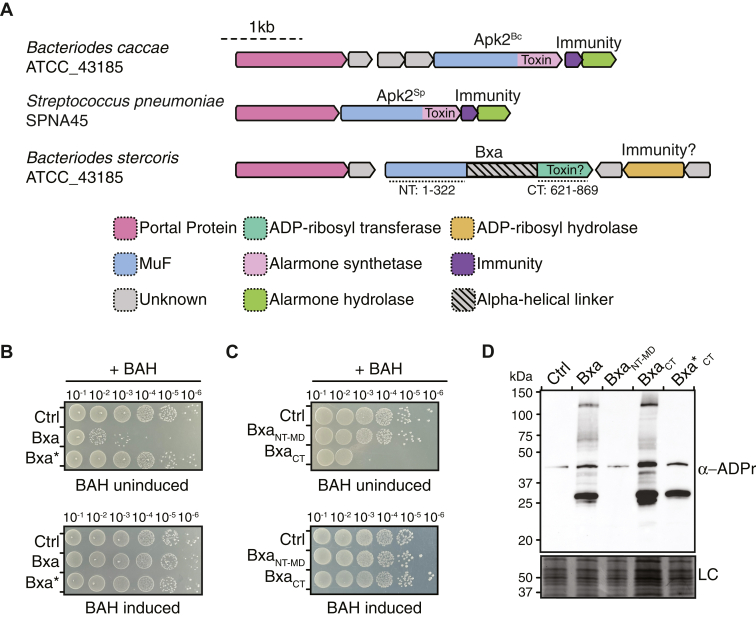
**Protein ADP-ribosylation by Bxa results in bacterial cell death**. *A*, schematic of gene loci encoding Apk2 family proteins (Apk2^Bc^ and Apk2^Sp^) and Bxa. The conserved N-terminal MuF domains of Apk2^Bc^, Apk2^Sp^, and Bxa are colored *purple* whereas their variable C-terminal toxin domains are coloured by function: ADP-ribosyltransferase (*green*) and alarmone synthetase (*purple*). Each Apk2 protein is encoded alongside an alarmone hydrolase (*pink*) whereas Bxa is encoded alongside a predicted ADP-ribosyl hydrolase BACSTE_RS09445 (BAH; *yellow*) and two additional small open reading frames of unknown function, BACSTE_RS09440 and BACSTE_RS09450 (*grey*). The Apk2 proteins are also encoded alongside a non-enzymatic immunity protein (*light green*). *B*, Tenfold serial dilutions of *E. coli* expressing either empty vector (Ctrl), full length Bxa or its catalytic mutant (Bxa∗) with or without co-expression of BAH (induced/uninduced). *C*, Ten-fold serial dilutions of *E. coli* expressing either empty vector (Ctrl), the N-terminal and middle domains of Bxa (Bxa_NT-MD_) as defined in (*A*), or the C-terminal toxin domain (Bxa_CT_) as delineated in (*A*) with or without co-expression of BAH (induced/uninduced). *D*, anti-ADPr immunoblot of total protein from *E. coli* cells expressing either empty vector (Ctrl), Bxa, or the indicated Bxa truncations. Non-specific bands from the SDS PAGE of these samples stained with Coomassie Brilliant Blue is used as a loading control (LC).

In addition to functioning as a host-cell targeting prophage encoded fitness factor implicated in *B. stercoris* ATCC 43185 gut colonization, Bxa’s similar genomic arrangement to the recently characterized antibacterial Apk2 MuF toxins led us to hypothesize that it may also possess antibacterial activity (Fig. 1*A*). Both Bxa and Apk2 are encoded in the head morphogenesis region of their respective prophage elements and contain N-terminal MuF domains (20, 21). Apk2’s C-terminal (p)ppApp synthetase activity is neutralized through the combined activities of a (p)ppApp hydrolase and a non-enzymatic immunity protein that physically blocks Apk2 catalytic activity (20, 21). Both immunity proteins are encoded adjacent to Apk2 suggesting that they may protect MuF toxin expressing cells from toxin-mediated death. Bxa is encoded alongside three open reading frames: BACSTE_RS09440, BACSTE_RS09445, and BACSTE_RS09450 (Fig. 1*A*). Of these genes, BACSTE_RS09445 is predicted to encode an ADP-ribosyl hydrolase, which typically function to catalyze the removal of ADP-ribose groups from ADP-ribosylated proteins (22). Based on this prediction, we henceforth refer to BACSTE_RS09445 as Bxa-associated ADP-ribosyl hydrolase (BAH). The remaining two ORFs, BACSTE_RS09440 and BACSTE_RS09450, lack homology to known bacteriophage head morphogenesis proteins and therefore may instead act as non-enzymatic immunity proteins to Bxa.

We began our investigations into the potential antibacterial activity of Bxa by attempting its expression in *E. coli*. Initially, we were unable to generate a *bxa*-encoding plasmid even though its expression was under the control of the highly repressible L-rhamnose promoter (23). We reasoned that the failure to generate this plasmid could be due to low levels of Bxa expression killing the heterologous host. Therefore, we next attempted to clone *bxa* into an *E. coli* strain expressing BAH from an IPTG-inducible promoter because this protein served as a likely immunity protein given its predicted ADP-ribosyl hydrolase activity. By propagating cells in the presence of IPTG, we were readily able to generate an expression plasmid harboring the *bxa* gene. Furthermore, we observed a substantial reduction in *E. coli* viability when cells were grown under non-BAH-inducing conditions indicating that in BAH’s absence, Bxa harbors antibacterial activity (Figs. 1*B* and S1*A*). To determine if this observed toxicity is due to the ADP-ribosyltransferase activity of Bxa, we next mutated its predicted catalytic glutamic acid to alanine (E820A, denoted as Bxa∗), which was previously shown to diminish its catalytic activity in *in vitro* assays, and examined the consequence of expressing this variant on *E. coli* growth (15). Consistent with its antibacterial properties being due to its ADP-ribosyltransferase activity, expression of Bxa∗ had no effect on *E. coli* viability (Figs. 1*B* and S1*A*). Collectively, these results indicate that the ADP-ribosyltransferase activity of Bxa is antibacterial and can be neutralized by BAH. Previously, Brown *et al.* suggested that the ART activity of Bxa arises from its C-terminal domain (residues 621–869; Bxa_CT_) so we additionally assessed whether this domain in isolation is sufficient to inhibit bacterial growth. Indeed, expression of Bxa lacking its C-terminal ADP-ribosyltransferase domain (residues 1–620; Bxa_NT-MD_) has no effect on *E. coli* growth whereas expression of the C-terminal ADP-ribosyltransferase domain, Bxa_CT_, exhibits similar levels of antibacterial activity as the full-length protein (Figs. 1*C* and S1*B*).

While cytoskeletal proteins within epithelial cells are targets for Bxa-mediated modification, our results indicate that bacterial proteins are likely also modified by the toxin. We directly assessed this by immunoblotting *E. coli* lysates following expression of either Bxa, Bxa_NT-MD_, Bxa_CT_, or Bxa∗_CT_ with an antibody that specifically recognizes ADP-ribose (ADPr) modifications and compared the α-ADPr signal to a lysate derived from non-intoxicated cells. The resulting Western blot revealed that intoxication by both Bxa and Bxa_CT_ results in the modification of many bacterial proteins, indicating that the toxin has a broad range of targets (Fig. 1*D*). By contrast, the α-ADPr signal from the non-toxic Bxa∗_CT_ expressing cells only identified a single high abundance ADP-ribosylated protein also present in cells expressing Bxa∗ suggesting that both proteins retain minimal ADP-ribosylation activity that does not compromise *E. coli* viability (Figs. 1*D* and S2). Although this modified band is also present in *E. coli* cells succumbing to the toxic activity of Bxa, we conclude that this modified species is not responsible for cell death because Bxa∗ expression is non-lethal. Taken together, our data show that the ADP-ribosyltransferase activity of Bxa promiscuously modifies many bacterial proteins leading to bacterial cell death.

### Bxa catalyzes the formation of N-linked ADP-ribose modifications

We next sought to enzymatically characterize the activity of Bxa. However, the expression of Bxa using high-copy expression vectors was lethal to our *E. coli* expression strain and could not be overcome by co-expressing BAH, leaving us unable to purify the wild-type form of the toxin for further biochemical analysis. Despite this technical challenge, we instead sought to identify which residue is targeted by Bxa for modification. To this end, we set out to purify a protein harboring Bxa-mediated ADP-ribosylation. Given Bxa’s promiscuous target range observed by α-ADPr immunoblotting, we reasoned that the toxin might be able to modify any target protein, even if not biologically relevant if it were to possess the requisite modification site(s) and be present at high enough concentration. We tested this reasoning by incubating cell lysate derived from *E. coli* expressing Bxa_CT_ or a Bxa-free control lysate with purified His_6_-Hcp2, a structural protein component of the T6SS apparatus from *Pseudomonas aeruginosa* (24). We chose Hcp2 as the target protein because of its stability following purification and its low likelihood of interacting with other proteins present in our *E. coli* overexpression strain due to it lacking a T6SS (24). Following incubation, we purified His_6_-Hcp2 from each lysate using nickel affinity chromatography. Using α-ADPr immunoblotting, we confirmed that only incubation with the Bxa-containing lysate results in the ADP-ribosylation of purified His_6_-Hcp2 (Hcp2-ADPr) indicating that the modification is specific to the activity of the toxin (Fig. S3*A*). Subsequent analyses with intact mass spectrometry revealed that the Hcp2-ADPr sample is heterogenous, containing both unmodified and modified protein, and that the latter harbours one detectable ADPr modification (Fig. S3*B*).

**Figure 2:**
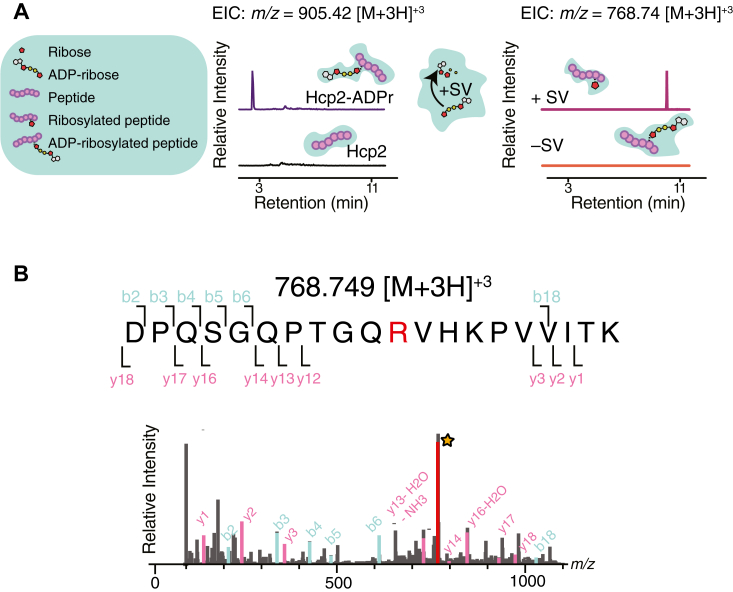
**Bxa targets arginine residues for ADP-ribosylation**. *A*, the extracted ion chromatogram (EIC) of mass spectrometry on modified Hcp2 (Hcp2-ADPr) and Hcp2 tryptic peptides (*left*). The EIC from mass spectrometry on Hcp2-ADPr tryptic peptides treated or untreated with snake-venom phosphodiesterase I (+/− SV). *B*, tandem mass spectrometry (MS/MS) of the *m/z* = 768.749 [M+3H]^+3^ corresponding to the ribosylated peptide of SV-treated ADPr-Hcp2 peptides.

To identify which residue contains the ADP-ribose moiety, we performed ESI-MS/MS peptide mapping on tryptic peptides of both Hcp2 and Hcp2-ADPr. This approach led to the identification of a unique species in the Hcp2-ADPr peptide sample (*m/z* = 905.42 [M+3H]^+3^) that was absent in the Bxa-free control sample. Treatment of the ADPr-Hcp2 sample with snake-venom phosphodiesterase I (SVPD), which cleaves the phosphodiesterase bond covalently linking the ribose to the adenosine diphosphate (ADP) nucleotide of ADPr leaving only the ribose group attached, resulted in loss of the *m/z* = 905.42 [M+H]^+3^ peak and the appearance of a new tryptic peptide, *m/z* = 768.74 [M+3H^3+^], consistent with the loss of the ADP (Fig. 2*A*). Tandem MS (MS/MS) of both *m/z* = 905.42 [M+H]^+3^ and *m/z* = 768.74 [M+3H^3+^] confirm that the species corresponds to the ribosylated (+152 Da) and ADP-ribosylated (+514 Da) form of the peptide **DPQSGQPTGQRVHKPVVITK** (Hcp2 residues 53–72), respectively. Following the identification of this peptide, we investigated which residue harbors the modification by further analysis of the MS/MS spectra. Fragments generated by MS/MS on the *m/z* = 768.74 [M+3H^3+^] species shortened the target peptide to a nine-residue sequence, **TGQRVHKPV**, that contains the modification (Fig. 2*B*). Within this shorter peptide, lysine and arginine are potential targets for N-linked modification whereas threonine is a potential target for O-linked modification. In the MS/MS on the *m/z* = 905.42 [M+3H]^+3^ peptide, we detected a species corresponding to ADP-ribosylated guanidine (*m/z* = 584.09), indicating that Arg63 is the site of modification (Fig. S3*C*). Altogether, the mass spectrometry analyses demonstrate that Bxa catalyzes formation of N-linked ADP-ribose adducts on arginine residues.

In an attempt to better understand how the activity of Bxa results in cellular intoxication, we also conducted post-translational modification-specific proteomics, which was previously used to successfully identify the protein targets of the bacteria-targeting T6SS ADP-ribosyltransferase toxin, Tre1 (25). However, despite following a similar protocol, our experiments did not result in the detection of modified protein targets (data not shown). Based on these results, we next compared the levels of ADP-ribosylation between cells intoxicated by Bxa *vs.* those intoxicated by Tre1 using α-ADPr immunoblot and found that the overall levels of ADP-ribosylation are much higher in Tre1 intoxicated cells compared to those expressing Bxa (Fig. S4), suggesting that levels of ADP-ribosylation by Bxa are sufficient for bacterial death but not for detection by modification-specific proteomics.

### Bxa activity can be neutralized by two distinct immunity proteins

The antibacterial activity of Bxa suggests that its expression in *B. stercoris* likely requires neutralization through dedicated immunity proteins. Our observation that BAH expression mitigates toxicity in *bxa-*encoding cells further supports this hypothesis. Thus, we next sought to further characterize BAH-mediated neutralization and identify whether BACTSTE_RS09440 or BACTSTE_RS09450 also function as immunity proteins for Bxa given their genomic proximity to the toxin.

**Figure 3:**
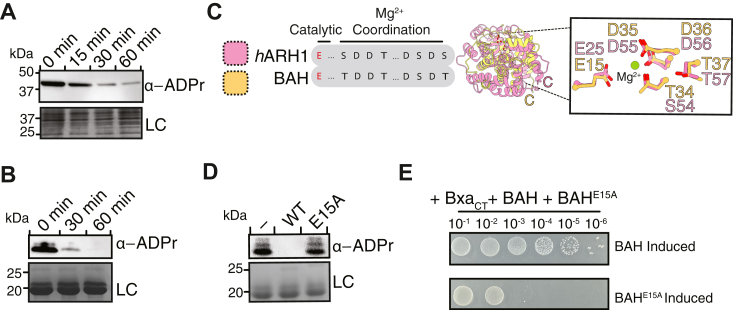
**An adjacently encoded ADP-ribosyl hydrolase protects against Bxa’s antibacterial activity.***A*, Anti-ADPr immunoblot from *E. coli* cells collected following 1 h of Bxa_CT_∗ expression (0 min) and following 15, 30, and 60 min of BAH induction. Non-specific bands from the SDS PAGE of these samples stained with Coomassie Brilliant Blue are used as a loading control (LC). *B*, anti-ADPr immunoblot of Hcp2-ADPr pre-incubation (0 min) with BAH and post-BAH incubation following 30 and 60 min. Hcp2 from the SDS PAGE of these samples stained with Coomassie Brilliant Blue is used as a loading control (LC). *C*, alignment of the AlphaFold three predicted structure of BAH (*yellow*) with the structure of *h*ARH1 (*pink*) (*right*) and the amino-acid sequence alignment of the proteins, highlighting shared catalytic and magnesium coordinating residues important for ADP-ribosyl hydrolase activity (*left*). *D*, anti-ADPr immunoblot of Hcp2-ADPr incubated with BAH (WT), BAH^E15A^ (E15A) or nothing (−) for 60 min. Hcp2 from the SDS PAGE of these samples stained with Coomassie Brilliant Blue is used as a loading control (LC). *E*, tenfold serial dilutions of *E. coli* encoding plasmid-borne Bxa_CT_ BAH, and BAH^E15A^ plated on media for the induction of either BAH (*top*) or BAH^E15A^ (*bottom*).

We first assayed whether BAH activity could lower the overall levels of ADP-ribosylation in intoxicated cells. To test this, BAH expression was induced following 1 h of Bxa_CT_∗ expression and ADPr levels were examined by α-ADPr immunoblotting. Bxa_CT_∗ was used for this experiment instead of Bxa_CT_ because it would allow ADP-ribosylated products to accumulate without causing cell death. In support of BAH functioning as an ARH, ADPr signal was lost in a time-dependent manner following BAH induction (Fig. 3*A*). This cell-based result was recapitulated *in vitro* as purified BAH similarly catalyzed the removal of ADPr from purified Hcp2-ADPr in a time-dependent manner (Fig. 3*B*). Comparing both the sequence and the predicted structure of BAH to that of human ARH1 (*h*ARH1; PDB 3HFW), a well-studied representative of the ARH1 sub-family that catalyzes the reversal of N-linked ADPr modifications, revealed that BAH possesses the key catalytic glutamic acid and magnesium-coordinating residues necessary for ARH1 activity (Figs. 3*C* and S5) (26, 27). In accordance with these two enzymes functioning similarly, mutation of the predicted catalytic glutamic acid in BAH to alanine (E15A) abrogated its ability to remove ADPr from Hcp2-ADPr (Fig. 3*D*). Using this same catalytic mutant, we also assessed whether the hydrolase activity of BAH is solely responsible for BAH-mediated Bxa neutralization. Using a three-plasmid co-expression system, *E. coli* harboring Bxa_CT_, BAH, and BAH^E15A^ were assessed for toxin-neutralization. With this system, we found that expression of wild-type BAH but not BAH^E15A^ allows for the growth of Bxa expressing *E. coli* (Figs. 3*E* and S1*C*), indicating that the hydrolase activity alone results in the neutralization of the toxin. Taken altogether, our work identifies BAH as an ARH1-like immunity protein of Bxa.

**Figure 4:**
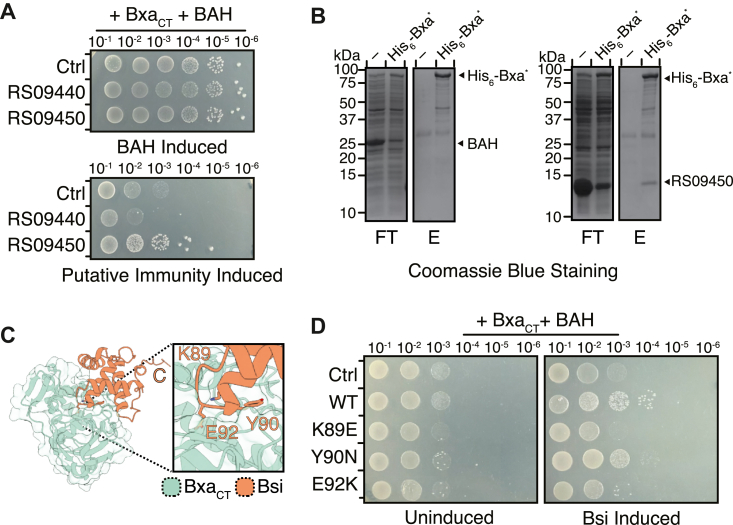
**A non-enzymatic immunity protein confers protection from Bxa *via* an inhibitory protein-protein interaction.***A*, tenfold serial dilutions of *E. coli* containing Bxa_CT_, BAH and either empty vector (Ctrl) or vector encoding either BACSTE_RS09440 (RS09440) or BACSTE_RS09450 (RS09450) plated on solid media. Serial dilutions were plated on media supplemented with an inducer for BAH (*top*) or the putative immunities (*bottom*). *B*, SDS PAGE of the flow-through (FT) and elution (E) from the nickel-affinity chromatography-based co-purification of His_6_-Bxa∗ with either untagged BAH (*left*) or untagged RS09450 (*right*) stained with Coomassie Brilliant Blue. *C*, modeling with AlphaFold three places Bsi (*orange*) in the active site of Bxa_CT_ (*green*). *D*, tenfold serial dilutions of *E. coli* containing Bxa_CT_, BAH, and empty vector (Ctrl) or vector encoding wild-type Bsi (WT), Bsi^K89E^ (K89E), Bsi^Y90N^ (Y90N), or Bsi^E92K^ (E92K) on plated solid media. Serial dilutions of *E. coli* were plated either on media with or without inducer for Bsi (Bsi induced/uninduced).

We considered that toxin neutralization could be additionally mediated by prohibitory protein-protein interactions as is observed for many toxin-immunity pairs (8, 21, 25, 28). For example, Apk2 is neutralized by Aph1, a (p)ppApp hydrolase that reverses alarmone synthesis, but also by BcTis1 which prevents alarmone synthesis by binding to the active site of the toxin (20, 21). This precedent led us to also investigate whether BACSTE_RS09440 or BACSTE_RS09450 could act as additional immunity proteins to Bxa given that both correspond to small gene products with no predicted enzymatic activity (Fig. S6). For this line of investigation, we used the previously described three-plasmid system to assay if the independent expression of either candidate immunity proteins would protect *E. coli* from Bxa-mediated toxicity in the absence of BAH. In doing so, we found that expression of BACSTE_RS09450 moderately neutralized the toxicity of Bxa whereas expression of BACSTE_RS09440 did not (Figs. 4*A* and S1*D*). Given that BACSTE_RS09450 is not predicted to encode an enzyme, we next assayed whether it physically interacts with Bxa. Using His_6_-Bxa∗, we assessed the interaction between the toxin and untagged BACSTE_RS09450 using nickel affinity chromatography and found that the two proteins co-purified with one another (Fig. 4*B*). By contrast, BAH, whose immunity function is not predicted to require a protein-protein interaction, did not co-purify with His_6_-Bxa∗ under the same experimental conditions. Based on these findings, we refer to BACSTE_RS09450 as Bxa structural immunity (Bsi).

To begin probing the protein-protein interaction interface between Bxa and Bsi, we next generated a model of the complex using AlphaFold 3. The resulting structural prediction places Bsi within the active site of Bxa’s ADP-ribosyltransferase domain, which is consistent with active site occlusion being the most prevalent mechanism of immunity inhibition observed for antibacterial toxins (Fig. S7) (28, 29, 30, 31, 32). However, the interface predicted template modeling (iPTM) score for this structural model (0.77) only slightly exceeds the recommended threshold based on experimentally confirmed protein-protein interaction interfaces (>0.75) and we therefore sought to validate this prediction using site-specific mutagenesis (33). To this end, we first used PDBePISA to predict which residues have the greatest thermodynamic contribution to complex formation. This analysis identified residues Lys89, Tyr90, and Glu92 as important mediators of Bsi’s ability to bind to and inhibit Bxa’s toxic catalytic activity (Fig. 4*C* and Tables S1, and S2). We mutagenized each of these residues to an amino acid with substantially different chemical properties (Lys89Glu, Tyr90Asn, Glu92Lys) and examined the ability of the resulting variants to neutralize Bxa-mediated toxicity when co-expressed in *E. coli*. In doing so, we found that both Bsi^K89E^ and Bsi^E92K^ are unable to neutralize Bxa whereas Bsi^Y90N^ was protective to levels comparable to the wild-type immunity protein (Figs. 4*D* and S1*E*). Altogether, these results suggest a model where Bxa’s ADP-ribosyltransferase activity is reversed enzymatically by BAH and inhibited through a physical interaction with Bsi.

### Bxa is a member of a prophage-encoded polymorphic toxin family

**Figure 5:**
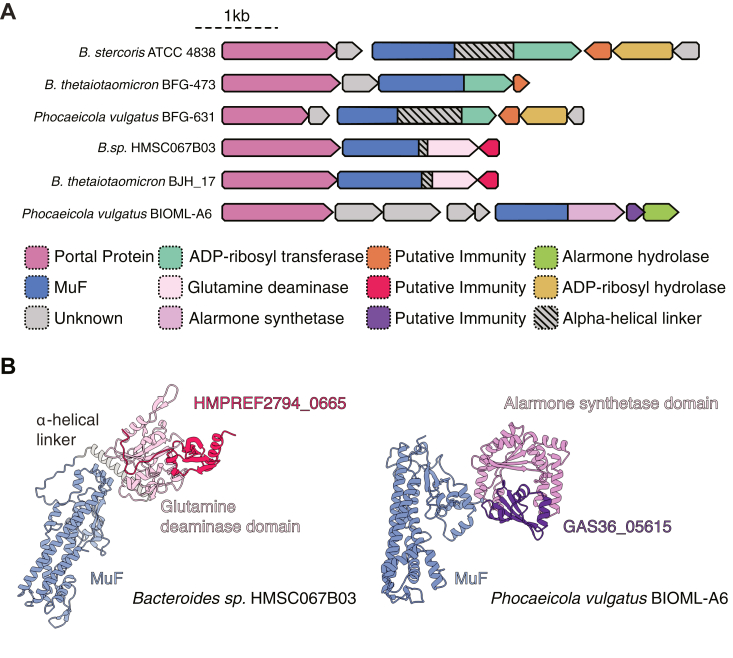
**Homologs of Bxa’s N-terminal MuF domain are encoded by diverse prophages and are fused to various C-terminal toxin domains with predicted antibacterial activity**. *A*, a schematic diagram of Bxa and selected Bxa_NT_ homologs, grouped by their C- terminal domains. *B*, alphaFold three modeling of the predicted glutamine deaminase homolog of Bxa (*light-pink*) in complex with its predicted immunity protein (*dark-pink*) (*left*) and the predicted alarmone synthetase homolog of Bxa (*light-purple*) in complex with its predicted immunity protein (*dark-purple*).

Our results thus far demonstrate that much like the recently characterized Apk2 toxins, Bxa possesses antibacterial properties that can be mitigated by the co-expression of adjacently encoded immunity proteins (20, 21). Given that previous sequence-based techniques identified a diverse toxin family associated with the so-called MuF domain, we wanted to investigate whether homologs of the N-terminal MuF domain of Bxa are found fused to other antibacterial toxins besides predicted ADP-ribosyltransferases (8, 19). Indeed, by performing a BLASTp search against the non-redundant sequence database using the N-terminal domain of Bxa as a search query (residues 1–322, Bxa_NT_), we identified homologous proteins throughout the order *Bacteroidales* that not only contain predicted ADP-ribosyltransferase domains like Bxa, but also toxic glutamine deaminase and Apk2-like alarmone synthetase domains (Fig. 5*A*) (8, 20, 21, 34). Furthermore, each identified MuF polymorphic toxin is followed by at least one small ORF that we speculate functions as a non-enzymatic immunity protein. In the case of the identified alarmone synthetase homolog, we find a predicted Aph1-like alarmone hydrolase adjacent to the toxin, much like the arrangement observed for the Apk2 toxins (20, 21). This overall gene organization suggests that the identified homologs with C-terminal toxin extensions likely also require neutralization by immunity proteins. To probe this prediction further, we examined a representative glutamine deaminase toxin from *Bacteroides* sp. (WP_139147237.1) and a representative alarmone synthetase from *Phocaeicola vulgatus* (KAB3885718.1) and performed gene neighbourhood analyses using the webFlaGs server (35). In both cases, we found that homologs of the adjacently encoded ORFs are always found with their respective MuF domain-containing toxin (Fig. S8, *A* and *B*). Additionally, structural modeling of each small ORF with its respective toxin using AlphaFold three predicts a high-confidence complex in which the putative immunity protein interacts with the predicted active site of each toxin (Figs. 5*B* and S9, *A* and *B*). In conclusion, our ability to identify diverse prophage encoded toxin domains using Bxa_NT_ as a search query suggests that Bxa belongs to a broader family of polymorphic toxins encoded within prophage elements.

## Discussion

In the present study, we show that Bxa, an ADP-ribosyltransferase involved in *B. stercoris* gut colonization, is also toxic to bacteria and requires neutralization by the immunity proteins BAH and Bsi to prevent self-intoxication. Orthologs of *bxa* co-occur with BAH and Bsi-like encoding genes in diverse human gut *Bacteroidales*, indicating that the observed antibacterial activity is likely a conserved feature of Bxa-like proteins. We also found that BACSTE_RS09440, encoded in the same cluster as Bsi and BAH, is not able to neutralize Bxa-mediated toxicity. While the function of this protein remains unknown, both its small size and genomic context suggest it too functions as an immunity protein. In other polymorphic toxin systems, polyimmunity loci have been identified and often encode immunity proteins in the absence of a corresponding toxin (8, 36, 37, 38, 39, 40). Therefore, it may be that MuF-associated toxin gene clusters also harbor additional immunity proteins that protect against non-cognate toxins.

It is unclear why Bxa targets the cytoskeletal components of epithelial cells while also exhibiting toxicity within the bacterial cytoplasm. It may be that the enzymatic promiscuity of Bxa results in the modification of essential bacterial proteins upon its expression in toxin-producing *Bacteroides* cells thus necessitating its neutralization by immunity proteins prior to secretion. A similar phenomenon has been observed for the eukaryotic-targeting NAD ^+^ glycohydrolase toxins *Streptococcus pyogenes* NAD^+^-glycohydrolase (SPN) and *Mycobacterium tuberculosis* necrotizing toxin (TNT), whose activities facilitate pathogenesis and host colonization, respectively, but can also deplete NAD^+^ in the toxin-producing bacteria (30, 41, 42, 43, 44). Consequently, although SPN and TNT play no known role in bacterial antagonism, each toxin is encoded alongside and neutralized by a cognate immunity protein that prevents self-intoxication during toxin production and export (30, 41, 44). There are also examples of antibacterial toxins that additionally target eukaryotic cells such as the T6SS phospholipase effector PldB from *P. aeruginosa*, which not only mediates T6SS-dependent interbacterial killing but also epithelial cell invasion during pathogenesis by degrading phosphatidylethanolamine (PE), a component of both eukaryotic and bacterial membranes (45, 46, 47). Therefore, it may be that the host cell-targeting activity of Bxa is only one facet of its function and the modification of bacterial proteins in competitors could also drive the fitness observed in *bxa* encoding ΦBxa lysogens (15). Notably Bxa is unlike SPN, TNT, and PldB in that its activity does not antagonize eukaryotic cells but instead supports host colonization by driving the release of digestible nucleosides, which suggests cooperation between the ΦBxa lysogen and the non-phage related ribose-utilization system(s) of *B. stercoris* (14, 15, 30, 42, 44, 47).

Our informatics analyses indicate that Bxa is a member of the previously described bacteriophage-encoded MuF toxin family, which is perhaps not surprising given Bxa’s shared genomic organization with the Apk2 family of (p)ppApp synthetases (8, 19, 20, 21). Interestingly, whereas Bxa has been shown to exert its enzymatic activity in both eukaryotic and prokaryotic cells, the activity of the only other MuF member, Apk2, has only been investigated in the context of its ability to dysregulate bacterial physiology (20, 21, 29, 48). While the shared genomic arrangement of these toxins suggest they play biologically similar roles, it remains to be investigated whether the Apk2 and other MuF associated toxins mediate host colonization.

## Experimental procedures

### Bacterial strains and culturing conditions

All bacterial strains used in this study can be found in Table S3. *E. coli* strains were grown in Lennox lysogeny (LB) broth (10 g/ml NaCl, 10 g/ml tryptone, 5 g/ml yeast extract) with aeration at 37 °C. Where appropriate, cultures were supplemented with 15 μg/ml gentamicin, 150 μg/ml carbenicillin, 100 μg/ml ampicillin, 200 μg/ml trimethoprim, 25 μg/ml chloramphenicol, 500 μM IPTG, 0.2% w/v L-rhamnose, and 0.2% w/v L-arabinose. *E. coli* XL1-Blue was used for plasmid maintenance and toxicity experiments. *E. coli* BL21 (DE3) CodonPlus was used for protein expression and purification of BAH and Hcp2. *E. coli* BL21 (DE3) pLysS was used for protein expression and co-purification with all pETDuet-1 constructs.

### Plasmid construction and expression

All plasmids used in this study can be found in Table S4. All primers were synthesized and purified by Integrated DNA technologies (IDT). Phusion polymerase, restriction enzymes, and T4 DNA ligase were obtained from New England Biolabs (NEB). Sanger sequencing was performed by TCAG Sequencing and Genewiz Incorporated. Plasmids used for heterologous expression include pETDuet-1, pET29b, pBAD33, pSCrhaB2-CV, and pPSV39-CV. All expression plasmids were constructed using standard restriction enzyme-based cloning procedures.

### Bacterial toxicity assays

Due to its toxicity, we were unable to generate a strain containing a *bxa* or *bxa*_*CT*_ encoding plasmid alone. To overcome this, *bxa* and *bxa*_*CT*_ were ligated into pSCrhaB2-CV and transformed into chemically competent *E. coli* XL1-Blue cells expressing BAH cloned into pPSV39-CV. The same cloning strategy was taken to generate point mutants and truncations of Bxa. All *E. coli* XL1-Blue strains used for toxicity assays were grown for 18 h at 37 °C in LB broth supplemented with 500 μM IPTG and the appropriate antibiotics (15 μg/ml gentamicin, 200 μg/ml trimethoprim, and/or 25 μg/ml chloramphenicol).

For spot plate assays, overnight cultures were serially diluted 10-fold and 10 μl of each dilution was plated on solid LB media supplemented with 500 μM IPTG or 0.2% L-arabinose for growth restoration and the appropriate antibiotics (15 μg/ml gentamicin, 200 μg/ml trimethoprim, and/or 25 μg/ml chloramphenicol). For uninduced conditions, 10 μl of each dilution was plated on solid LB media supplemented with the appropriate antibiotics (15 μg/ml gentamicin, 200 μg/ml trimethoprim, and/or 25 μg/ml chloramphenicol). Spots were left to dry, and plates were then placed in the 37 °C incubator for growth overnight.

For the isolation of intoxicated cells, stationary overnight cultures were diluted 1:50 into fresh LB broth supplemented with IPTG and the appropriate antibiotics. Tre1_Tox_ cells were not grown in the presence of IPTG. Cells were grown at 37 °C shaking until an OD_600_ of 0.4 to 0.6 was reached. Toxin expression was induced following the addition 0.2% w/v L-rhamnose and cells were collected through centrifugation at 7800*g* for 10 min following 30 min of intoxication. Cell pellets were resuspended in 1× PBS and mixed 1:1 with 2× SDS-loading buffer for analysis by SDS PAGE and subsequent western blotting.

### Protein purification

For protein purification, 50 ml overnight stationary cultures harbouring expression vectors (pET29b::BAH, pET29b::BAH^E15A^, pETDuet-1::Bxa::Bsi, pETDuet-1::Bxa::BAH and pET29b::Hcp2) were diluted 1:50 into 1 L of fresh LB broth supplemented with the appropriate antibiotic and grown at 37 °C until an OD_600_ of 0.6 was reached. The growth temperature was then decreased to 18 °C, upon which protein expression was induced with 1 mM IPTG. Induced cells were grown for an additional 12 to 18 h. Following induction, cells were pelleted and either subject to lysis or frozen for future use.

Cell pellets were resuspended in lysis buffer (50 mM Tris-Hcl pH 8, 500 mM NaCl, 5 mM 2-mercaptoethanol, and 5 mM imidazole) prior to lysis by sonication (amplitude 35%, 30 s pulses for 2 min per sample). After sonication, the resulting lysates were cleared by centrifugation at 39,000*g* for 60 min. The soluble fraction of the clear lysate was then applied to a gravity flow column containing 1.5 ml of Ni^2+^-nitrilotriacetic acid (Ni-NTA) agarose pre-equilibrated with lysis buffer. The column was then washed three times with five column volumes of lysis buffer. Bound proteins were eluted with lysis buffer supplemented with 400 mM imidazole. His_6_-BAH and His_6_-BAH^E15A^ were further purified by fast-protein liquid chromatography (FPLC) using gel filtration on a HiLoad 16/600 Superdex 200 column equilibrated in 20 mM Tris-HCl pH 8.0, 150 mM NaCl and 2 mM dithiothreitol.

### ADP-ribosylation of purified Hcp2

Stationary phase cultures of either *E. coli* XL1-Blue containing pSCrhaB2-CV::Bxa_CT_ and pPSV39-CV::BAH or *E. coli* XL1-Blue containing empty vectors were diluted 1:50 in 1 L of LB broth and grown at 37 °C. Upon reaching an OD_600_ of 0.6, 0.2% w/v L-rhamnose was supplemented into the media, and cells were grown for an additional 30 min to prior to being collected by centrifugation as described earlier. Pelleted cells were then resuspended in lysis buffer (50 mM Tris-Hcl pH 8, 500 mM NaCl, and 5 mM imidazole) and lysed by sonification (amplitude 35%, 30 s pulses for 2 min per sample). The soluble fraction was collected from lysate cleared by centrifugation as described earlier. To this, purified Hcp2 and 100 μM of NAD^+^ were added to each lysate and incubated shaking at room temperature for 1 h. This lysate was then applied to a gravity flow column containing 1.5 ml Ni-NTA agarose pre-equilibrated in lysis buffer. Following three washes with five column volumes of lysis buffer, bound Hcp2 was re-purified as described above and frozen at −80 °C for storage.

### Proteomics

Preparation of peptides was performed as described by Ting *et al.* (2018) with minor variations. Briefly, cell pellets of either intoxicated or empty vector-expressing cells were collected as described above and resuspended in 100 mM ammonium bicarbonate and 8M urea. The cell suspensions were then sonicated (amplitude 35%, 10 s pulses for 2 min per sample), and the lysate cleared of cellular debris through centrifugation for 15 min at 16,000*g* in 4 °C. Protein samples were normalized to 200 μg and reduced with 5 mM TCEP for 1 h at 37 °C, followed by alkylation with 10 mM iodoacetamide for 30 min at 25 °C in the dark. Alkylation was then quenched by the addition of 12 mM N-acetyl cysteine. Samples were then diluted with 100 mM ammonium bicarbonate to lower the urea concentration to 1.5 M and incubated overnight with 10 μg of sequencing grade trypsin (Promega) at 37 °C. Peptides were dried *via* centrifugation at 60 °C using a vacuum centrifuge (Vacufuge Plus) and resuspended in 5% acetonitrile (ACN) 0.1% formic acid (FA) pH 2. Samples were loaded onto a Sep-pak C18 column activated with two 100% ACN washes and equilibrated with a 50% ACN wash. Following sample application, the cartridge was washed twice with 5% ACN 0.1% FA pH 2. Samples were eluted with 80% ACN 0.1% FA pH two and dried *via* centrifugation at 60 °C with the Vacufuge Plus.

Peptides were reconstituted with 0.1% FA in water and injected onto a Neo trap cartridge coupled with an analytical column (75 μm ID × 50 cm PepMap Neo C18, 2 μm). Samples were separated using a linear gradient of solvent A (0.1% formic acid in water) and solvent B (0.1% formic acid in ACN) over 120 min using a Vanquish Neo UHPLC System coupled to an Orbitrap Eclipse Tribrid Mass Spectrometer with FAIMS Pro Duo interface (Thermo Fisher Scientific). The resulting tandem MS data was queried for protein identification against the UniProt *E. coli* K-12 database using Mascot v.2.8.3 (Matrix Science). The following modifications were set as search parameters: peptide mass tolerance at 10 ppm, trypsin enzyme, three allowed missed cleavage sites, cysteine carbamidomethylation (static modification), and methionine oxidation, asparagine/glutamine deamination, arginine ADP-ribosylation, and protein N-term acetylation (variable modification). The search results were validated with 1% FDR of protein threshold and 90% of peptide threshold using Scaffold v5.3.0 (Proteome Software).

### Western blotting

To analyze the ADP-ribosylated proteins modified by Bxa, 2 ml cultures of *E. coli* XL1-Blue expressing either full-length Bxa, truncated Bxa (Bxa_NT-MD_, Bxa_CT_), or empty vector were pelleted by centrifugation at 7800*g* after 30 min of toxin expression. Cell pellets were resuspended in 40 μl of 1× PBS, mixed with 2× SDS-loading buffer, and boiled at 95 °C for 10 min. After boiling, samples were centrifuged at 21,000*g* and equal volumes were loaded onto a 12% SDS PAGE gel. The gel was first run for 20 min at 85 V, followed by 40 min at 180 V. Total protein was then wet transferred onto a nitrocellulose membrane using the Mini Trans-Blot electrophoretic system (BioRad). The transfer was run at 100 V for 30 min, after which the membrane was blocked in PBS-T with 0.2% w/v I-Block solution for 1 h shaking gently at room temperature. Primary antibody (α-ADPr) was added at a 1:2500 dilution and incubated overnight shaking gently at 4 °C. Following incubation with the primary antibody, the membrane was washed with PBS-T and secondary antibody (α-Rabbit) was added and left to incubate with the blot for 1 h at room temperature, shaking gently. Following another wash with PBS-T, the blot was subsequently developed using Clarity Max ECL substrate (BioRad) and visualized using a ChemiDoc instrument (BioRad).

### BAH enzymatic assays

To measure the activity of BAH *in vivo*, stationary phase cultures of *E. coli* XL1-Blue containing pSCrhaB2-CV::Bxa∗_CT_ and pPSV39-CV::BAH were diluted 1:50 into fresh LB broth. This culture was grown at 37 °C until it reached an OD_600_ of 0.5, upon which 0.2% w/v L-rhamnose was added to induce expression of Bxa∗_CT_. After 1 h of toxin expression, IPTG was added to a concentration of 500 μM to induce the expression of BAH, and 1 ml cell samples were collected at 0 min (pre-addition of IPTG), 15 min, 30 min, and 60 min post-addition of IPTG. Upon collection, cell samples were first normalized to the OD_600_ of the 0-min time point and then pelleted by centrifugation as described above. Cell pellets were then resuspended in 20 μl of 1× PBS, mixed with 2× SDS-loading buffer, and boiled at 95 °C for 10 min. The resulting samples were analyzed *via* western blotting as previously described.

To measure the ability of BAH to reverse the modification on Hcp2-ADPr, 10 μM of purified BAH in ADP-ribosyl hydrolysis buffer (20 mM Tris-HCl pH 8, 50 mM NaCl, 5 mM MnCl_2_, and 5 mM MgCl_2_) was added to modified Hcp2 and incubated for 1 h at 37 °C. During this incubation, time-points were removed prior to and at 30- and 60-min post-addition of BAH and diluted 1:1 in 2× SDS-loading buffer to quench the ADP-ribosyl hydrolase reaction. These samples were then boiled at 95 °C for 10 min prior to analysis by SDS PAGE and western blotting. For samples treated with purified BAH^E15A^, only the 60-min time point was analyzed.

### Liquid chromatography electrospray ionization-mass spectrometry (LC ESI-MS)

Intact protein ESI-MS and peptide mapping were conducted on the Agilent 6546 LC/Q-ToF. All columns were equilibrated with a mobile phase of solvent A (0.1% formic acid in water) and separated with solvent B (0.1% formic acid and 2% acetonitrile in water). Following the injection of 3.00 μl of the sample, the sample was monitored for a range of 100 to 1700 (*m/z*) at one spectra/sec in positive ion mode and the elution was monitored with full-spectrum UV absorbance. For peptide mapping of Hcp2, both Hcp2 and Hcp2-ADPr were treated with 5 μg of sequencing-grade trypsin (Promega) overnight. The following peptides were analyzed using an analytical column (Agilent EclipsePlus C18 2.1 × 100 mm, 1.8 μm) with the column oven set to 30 °C. Tryptic peptides were separated with a gradient of solvent B starting at 2% to 95% over 15 min, and MS2 scans were collected between 0.5 and 12 min. Intact protein ESI-MS of Hcp2 and Hcp2-ADPr was conducted using an analytical column (Zorbax 300SB C3 3.0 × 150 3.5, μm) with the column oven set to 65 °C. The column was equilibrated with solvent A and proteins were separated with a gradient of solvent B starting at 5% to 60% over 30 min.

All spectra were collected with MassHunter 10.1 Data Acquisition and analyzed with MassHunter 10.0 Qualitative Analysis and Mmass 3 (49). Intact-MS spectra were deconvoluted using Unidec (50).

### Identification of bxa homologs

A BLASTp search was performed against the non-redundant (nr) protein database using the N-terminal domain of Bxa, defined by its predicted structure, as the search query (34). A list of the accession numbers from the subsequent hits was compiled and filtered for sequences with over 75% query cover, and from this list representative sequences were selected to display the polymorphism of the C-terminal domain. These are available in the supporting information. Domain boundaries were determined based on the structural predictions present in either the AlphaFold three database or based on structures modelled by AlphaFold 3 (51).

### Gene neighborhood analysis

A BLASTp search was performed against the RefSeq protein database with the open reading frames of interest (34). An accession list was compiled from each flanking gene analysis to include all hits that were found to be encoded close to a bacteriophage portal protein gene. This accession list was submitted to webFlaGs for genetic neighborhood analysis (35). A representative of each gene arrangement was then compiled using Clinker (52). To ascertain the function of unknown genes, the amino acid sequence of each gene was submitted to BLASTp, HHpred the InterPro server (34, 53, 54, 55, 56).

### Structural modeling

All protein models were visualized with ChimeraX v1.4 (57).

### Protein alignment

All protein-protein alignments and resulting RMSD calculations were conducted using PyMOL Molecular Graphics System, Version 3.0 Schrödinger, LLC (58).

## Data availability

All data are present in the main manuscript and supporting information.

## Supporting information

This article contains supporting information (15, 23, 28, 59, 60).

## Conflict of interests

The authors declare that they have no conflicts of interest with the contents of this article. The author is an Editorial Board Member/Editor-in-Chief/Associate Editor/Guest Editor for the *Journal of Biological Chemistry* and was not involved in the editorial review or the decision to publish this article.

## References

[1] Richard M.L., Sokol H. (2019). The gut mycobiota: insights into analysis, environmental interactions and role in gastrointestinal diseases. Nat. Rev. Gastroenterol. Hepatol..

[2] Gerrick E.R., Zlitni S., West P.T., Carter M.M., Mechler C.M., Olm M.R. (2024). Metabolic diversity in commensal protists regulates intestinal immunity and trans-kingdom competition. Cell.

[3] Mafra D., Ribeiro M., Fonseca L., Regis B., Cardozo L.F.M.F., Fragoso Dos Santos H. (2022). Archaea from the gut microbiota of humans: could be linked to chronic diseases?. Anaerobe.

[4] The Human Microbiome Project Consortium (2012). Structure, function and diversity of the healthy human microbiome. Nature.

[5] Wexler A.G., Goodman A.L. (2017). An insider's perspective: Bacteroides as a window into the microbiome. Nat. Microbiol..

[6] Russell A.B., Wexler A.G., Harding B.N., Whitney J.C., Bohn A.J., Goo Y.A. (2014). A type VI secretion-related Pathway in Bacteroidetes mediates interbacterial antagonism. Cell Host Microbe.

[7] Chatzidaki-Livanis M., Geva-Zatorsky N., Comstock L.E. (2016). Bacteroides fragilis type VI secretion systems use novel effector and immunity proteins to antagonize human gut Bacteroidales species. Proc. Natl. Acad. Sci. U. S. A..

[8] Zhang D., de Souza R.F., Anantharaman V., Iyer L.M., Aravind L. (2012). Polymorphic toxin systems: comprehensive characterization of trafficking modes, processing, mechanisms of action, immunity and ecology using comparative genomics. Biol. Direct.

[9] Coyne M.J., Roelofs K.G., Comstock L.E. (2016). Type VI secretion systems of human gut Bacteroidales segregate into three genetic architectures, two of which are contained on mobile genetic elements. BMC Genomics.

[10] Verster A.J., Ross B.D., Radey M.C., Bao Y., Goodman A.L., Mougous J.D., Borenstein E. (2017). The landscape of type VI secretion across human gut microbiomes reveals its role in community composition. Cell Host Microbe.

[11] Schwalm N.D., Groisman E.A. (2017). Navigating the gut buffet: control of polysaccharide utilization in Bacteroides spp. Trends Microbiol..

[12] Xu J., Bjursell M.K., Himrod J., Deng S., Carmichael L.K., Chiang H.C. (2003). A genomic View of the human-Bacteroides thetaiotaomicron symbiosis. Science.

[13] Cuskin F., Lowe E.C., Temple M.J., Zhu Y., Cameron E., Pudlo N.A. (2015). Human gut Bacteroidetes can utilize yeast mannan through a selfish mechanism. Nature.

[14] Glowacki R.W.P., Pudlo N.A., Tuncil Y., Luis A.S., Sajjakulnukit P., Terekhov A.I. (2020). A ribose-scavenging system confers colonization Fitness on the human gut symbiont Bacteroides thetaiotaomicron in a diet-specific manner. Cell Host Microbe.

[15] Brown E.M., Arellano-Santoyo H., Temple E.R., Costliow Z.A., Pichaud M., Hall A.B. (2021). Gut microbiome ADP-ribosyltransferases are widespread phage-encoded fitness factors. Cell Host Microbe.

[16] Manning K.A., Quiles-Puchalt N., Penadés J.R., Dokland T. (2018). A novel ejection protein from bacteriophage 80α that promotes lytic growth. Virology.

[17] Dröge A., Santos M.A., Stiege A.C., Alonso J.C., Lurz R., Trautner T.A., Tavares P. (2000). Shape and DNA packaging activity of bacteriophage SPP1 procapsid: protein components and interactions during assembl*y*. J. Mol. Biol..

[18] Bondy-Denomy J., Davidson A.R. (2014). When a virus is not a parasite: the beneficial effects of prophages on bacterial fitness. J. Microbiol..

[19] Jamet A., Touchon M., Ribeiro-Gonçalves B., Carriço J.A., Charbit A., Nassif X. (2017). A widespread family of polymorphic toxins encoded by temperate phages. BMC Biol..

[20] Ahmad S., Gordon I.J., Tsang K.K., Alexei A.G., Sychantha D., Colautti J. (2023). Identification of a broadly conserved family of enzymes that hydrolyze (p)ppApp. Proc. Natl. Acad. Sci. U. S. A..

[21] Bartoli J., Tempier A.C., Guzzi N.L., Piras C.M., Cascales E., Viala J.P.M. (2023). Characterization of a (p)ppApp synthetase belonging to a new family of polymorphic toxin associated with temperate phages. J. Mol. Biol..

[22] Mashimo M., Kato J., Moss J. (2014). Structure and function of the ARH family of ADP-ribosyl-acceptor hydrolases. DNA Repair (Amst).

[23] Cardona S.T., Valvano M.A. (2005). An expression vector containing a rhamnose-inducible promoter provides tightly regulated gene expression in Burkholderia cenocepacia. Plasmid.

[24] Allsopp L.P., Wood T.E., Howard S.A., Maggiorelli F., Nolan L.M., Wettstadt S., Filloux A. (2017). RsmA and AmrZ orchestrate the assembly of all three type VI secretion systems in Pseudomonas aeruginosa. Proc. Natl. Acad. Sci. U. S. A..

[25] Ting S.Y., Bosch D.E., Mangiameli S.M., Radey M.C., Huang S., Park Y.J. (2018). Bifunctional immunity proteins protect bacteria against FtsZ-targeting ADP-Ribosylating toxins. Cell.

[26] Moss J., Stanley S.J., Nightingale M.S., Murtagh J.J., Monaco L., Mishima K. (1992). Molecular and immunological characterization of ADP-ribosylarginine hydrolases. J. Biol. Chem..

[27] Takada T., Iida K., Moss J. (1993). Cloning and site-directed mutagenesis of human ADP-ribosylarginine hydrolase. J. Biol. Chem..

[28] Bullen N.P., Sychantha D., Thang S.S., Culviner P.H., Rudzite M., Ahmad S. (2022). An ADP-ribosyltransferase toxin kills bacterial cells by modifying structured non-coding RNAs. Mol. Cell.

[29] Ahmad S., Wang B., Walker M.D., Tran H.K.R., Stogios P.J., Savchenko A. (2019). An interbacterial toxin inhibits target cell growth by synthesizing (p)ppApp. Nature.

[30] Smith C.L., Ghosh J., Elam J.S., Pinkner J.S., Hultgren S.J., Caparon M.G., Ellenberger T. (2011). Structural basis of Streptococcus pyogenes immunity to its NAD+ glycohydrolase toxin. Structure.

[31] Beck C.M., Morse R.P., Cunningham D.A., Iniguez A., Low D.A., Goulding C.W., Hayes C.S. (2014). CdiA from Enterobacter cloacae Delivers a toxic ribosomal RNase into target bacteria. Structure.

[32] Wang Y., Zhou Y., Shi C., Liu J., Lv G., Huang H. (2022). A toxin-deformation dependent inhibition mechanism in the T7SS toxin-antitoxin system of Gram-positive bacteria. Nat. Commun..

[33] Yin R., Feng B.Y., Varshney A., Pierce B.G. (2022). Benchmarking AlphaFold for protein complex modeling reveals accuracy determinants. Protein Sci..

[34] Altschul S.F., Gish W., Miller W., Myers E.W., Lipman D.J. (1990). Basic local alignment search tool. J. Mol. Biol..

[35] Saha C.K., Sanches Pires R., Brolin H., Delannoy M., Atkinson G.C. (2021). FlaGs and webFlaGs: discovering novel biology through the analysis of gene neighbourhood conservation. Bioinformatics.

[36] Ross B.D., Verster A.J., Radey M.C., Schmidtke D.T., Pope C.E., Hoffman L.R. (2019). Human gut bacteria contain acquired interbacterial defence systems. Nature.

[37] Kirchberger P.C., Unterweger D., Provenzano D., Pukatzki S., Boucher Y. (2017). Sequential displacement of Type VI Secretion System effector genes leads to evolution of diverse immunity gene arrays in Vibrio cholerae. Sci. Rep..

[38] Barretto L.A.F., Fowler C.C. (2020). Identification of A Putative T6SS immunity islet in Salmonella Typhi. Pathogens.

[39] Garrett S.R., Mariano G., Dicks J., Palmer T. (2022). Homologous recombination between tandem paralogues drives evolution of a subset of type VII secretion system immunity genes in firmicute bacteria. Microb. Genom.

[40] Bowman L., Palmer T. (2021). The type VII secretion System of Staphylococcus. Annu. Rev. Microbiol..

[41] Tak U., Vlach J., Garza-Garcia A., William D., Danilchanka O., de Carvalho L.P.S. (2019). The tuberculosis necrotizing toxin is an NAD+ and NADP+ glycohydrolase with distinct enzymatic properties. J. Biol. Chem..

[42] Pajuelo D., Tak U., Zhang L., Danilchanka O., Tischler A.D., Niederweis M. (2021). Toxin secretion and trafficking by Mycobacterium tuberculosis. Nat. Commun..

[43] Bricker A.L., Cywes C., Ashbaugh C.D., Wessels M.R. (2002). NAD+-glycohydrolase acts as an intracellular toxin to enhance the extracellular survival of group A streptococci. Mol. Microbiol..

[44] Sun J., Siroy A., Lokareddy R.K., Speer A., Doornbos K.S., Cingolani G., Niederweis M. (2015). The tuberculosis necrotizing toxin kills macrophages by hydrolyzing NAD. Nat. Struct. Mol. Biol..

[45] Leoni Swart A., Laventie B.J., Sütterlin R., Junne T., Lauer L., Manfredi P. (2024). Pseudomonas aeruginosa breaches respiratory epithelia through goblet cell invasion in a microtissue model. Nat. Microbiol..

[46] Wood T.E., Howard S.A., Förster A., Nolan L.M., Manoli E., Bullen N.P. (2019). The Pseudomonas aeruginosa T6SS delivers a periplasmic toxin that disrupts bacterial cell morphology. Cell Rep..

[47] Jiang F., Waterfield N.R., Yang J., Yang G., Jin Q. (2014). A Pseudomonas aeruginosa type VI secretion phospholipase D effector targets both Prokaryotic and eukaryotic cells. Cell Host Microbe.

[48] Irving S.E., Choudhury N.R., Corrigan R.M. (2021). The stringent response and physiological roles of (pp)pGpp in bacteria. Nat. Rev. Microbiol..

[49] Strohalm M., Kavan D., Novák P., Volný M., Havlícek V. (2010). mMass 3: a cross-platform software Environment for precise Analysis of mass spectrometric data. Anal. Chem..

[50] Marty M.T., Baldwin A.J., Marklund E.G., Hochberg G.K.A., Benesch J.L.P., Robinson C.V. (2015). Bayesian deconvolution of mass and ion mobility spectra: from binary interactions to polydisperse ensembles. Anal. Chem..

[51] Abramson J., Adler J., Dunger J., Evans R., Green T., Pritzel A. (2024). Accurate structure prediction of biomolecular interactions with AlphaFold 3. Nature.

[52] Gilchrist C.L.M., Chooi Y.-H. (2021). Clinker & clustermap.js: automatic generation of gene cluster comparison figures. Bioinformatics.

[53] Zimmermann L., Stephens A., Nam S.Z., Rau D., Kübler J., Lozajic M. (2018). A completely reimplemented MPI bioinformatics Toolkit with a new HHpred Server at its core. J. Mol. Biol..

[54] Gabler F., Nam S.Z., Till S., Mirdita M., Steinegger M., Söding J. (2020). Protein sequence analysis using the MPI bioinformatics Toolkit. Curr. Protoc. Bioinform..

[55] Jones P., Binns D., Chang H.Y., Fraser M., Li W., McAnulla C. (2014). InterProScan 5: genome-scale protein function classification. Bioinformatics.

[56] Paysan-Lafosse T., Blum M., Chuguransky S., Grego T., Pinto B.L., Salazar G.A. (2023). InterPro in 2022. Nucleic Acids Res..

[57] Meng E.C., Goddard T.D., Pettersen E.F., Couch G.S., Pearson Z.J., Morris J.H., Ferrin T.E. (2023). UCSF ChimeraX: Tools for structure building and analysis. Protein Sci..

[58] DeLano W.L. (2002). Pymol: An open-source molecular graphics tool. CCP4 Newsl. Protein Crystallogr..

[59] Silverman J.M., Agnello D.M., Zheng H., Andrews B.T., Li M., Catalano C.E. (2013). Haemolysin coregulated protein is an exported receptor and chaperone of type VI secretion substrates. Mol. Cell.

[60] Chung H.S., Raetz C.R.H. (2010). Interchangeable domains in the Kdo transferases of Escherichia coli and Haemophilus influenzae. Biochemistry.

