# Supplementary figures and images for: The antibacterial activity of a prophage-encoded fitness factor is neutralized by two cognate immunity proteins
Source: J Biol Chem. 2024 Nov 16;300(12):108007. doi: 10.1016/j.jbc.2024.108007 (PMC11699363; doi:10.1016/j.jbc.2024.108007)

Figure S1

A

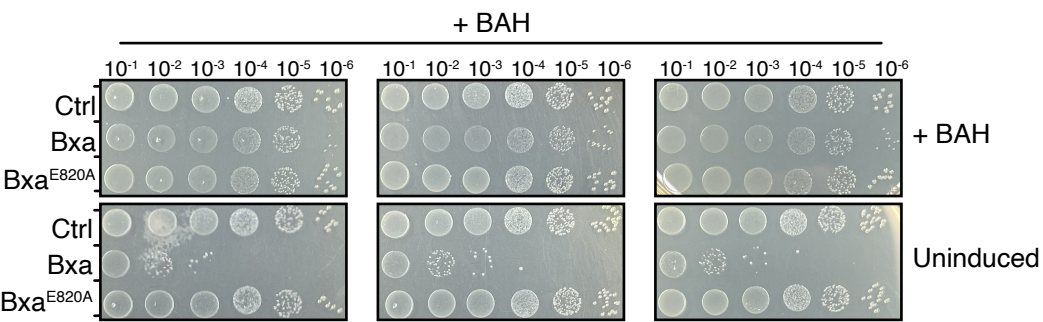

B

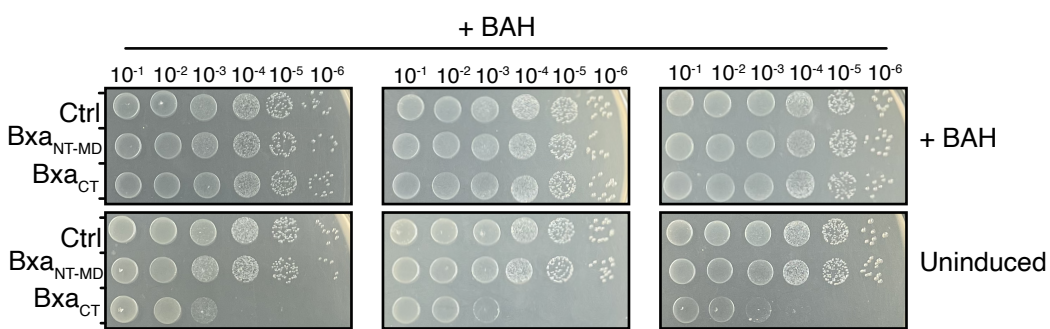

C

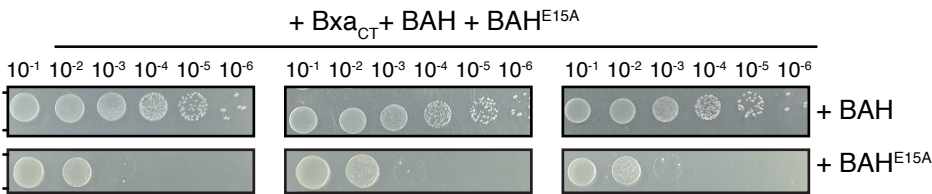

D

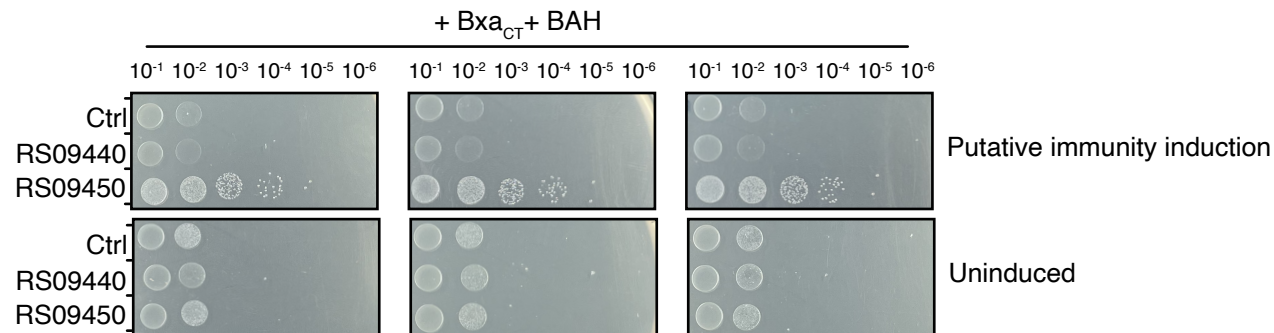

E

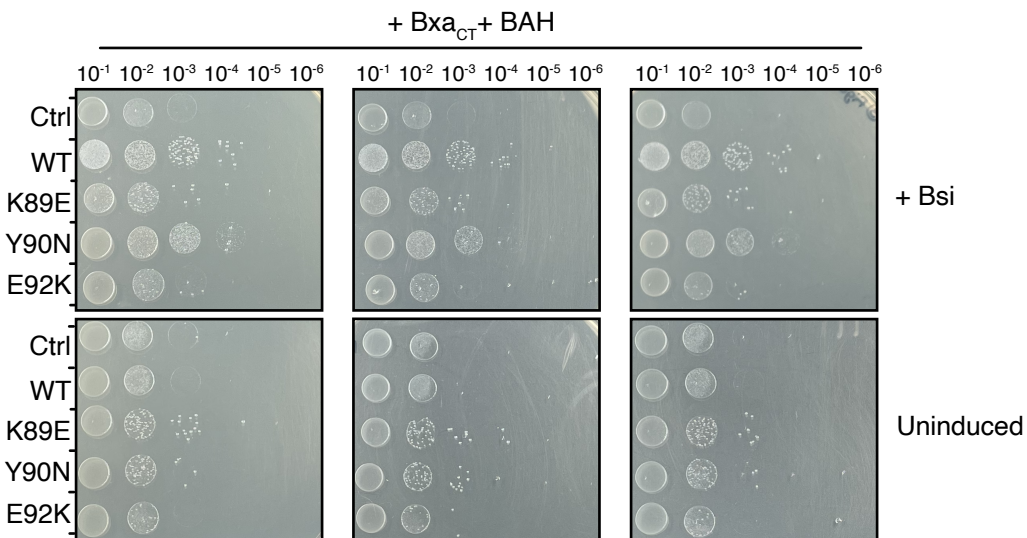

Supplement: Figure S1 [file mmc1.pdf]

Figure S2

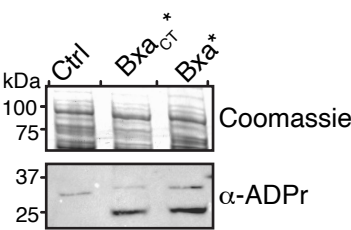

Supplement: Figure S2 [file mmc2.pdf]

Figure S3

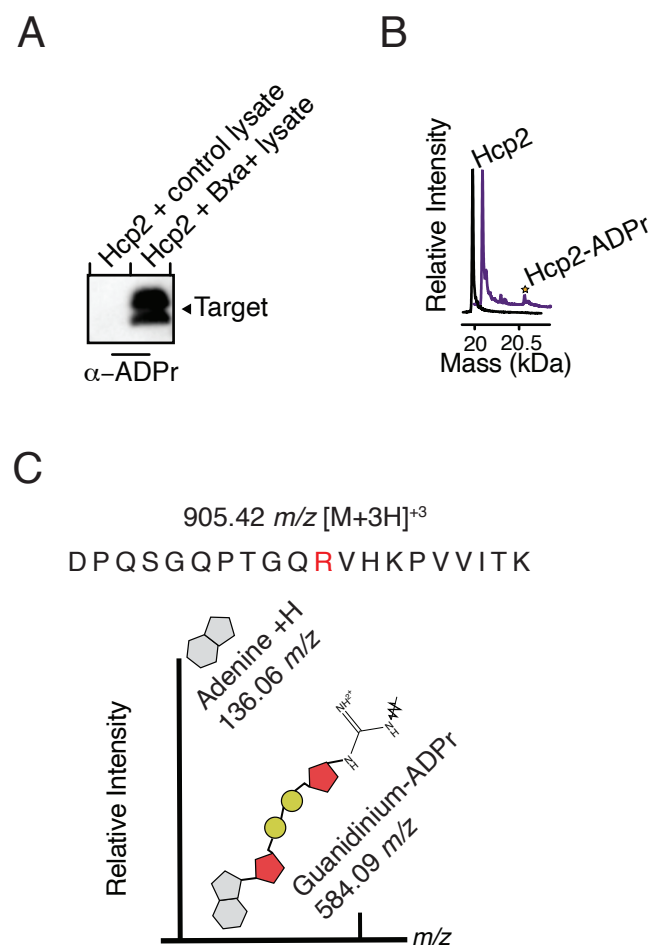

Supplement: Figure S3 [file mmc3.pdf]

Figure S4

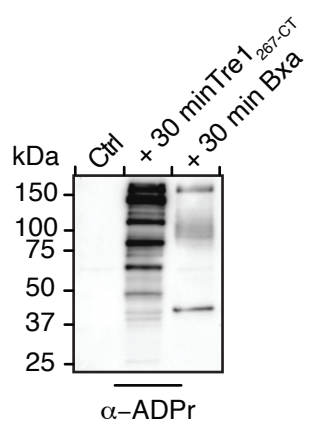

Supplement: Figure S4 [file mmc4.pdf]

Figure S5

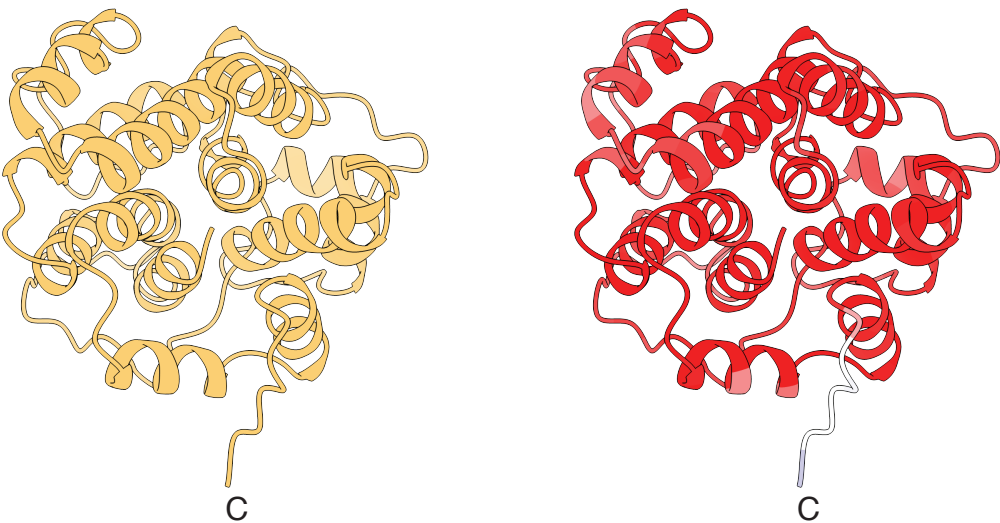

Supplement: Figure S5 [file mmc5.pdf]

Figure S6

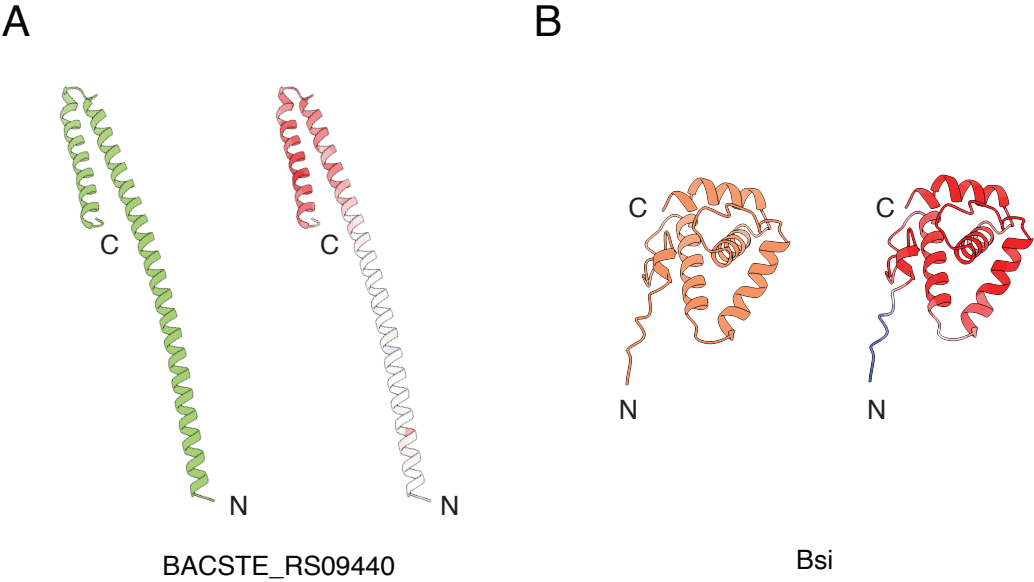

Supplement: Figure S6 [file mmc6.pdf]

Figure S7

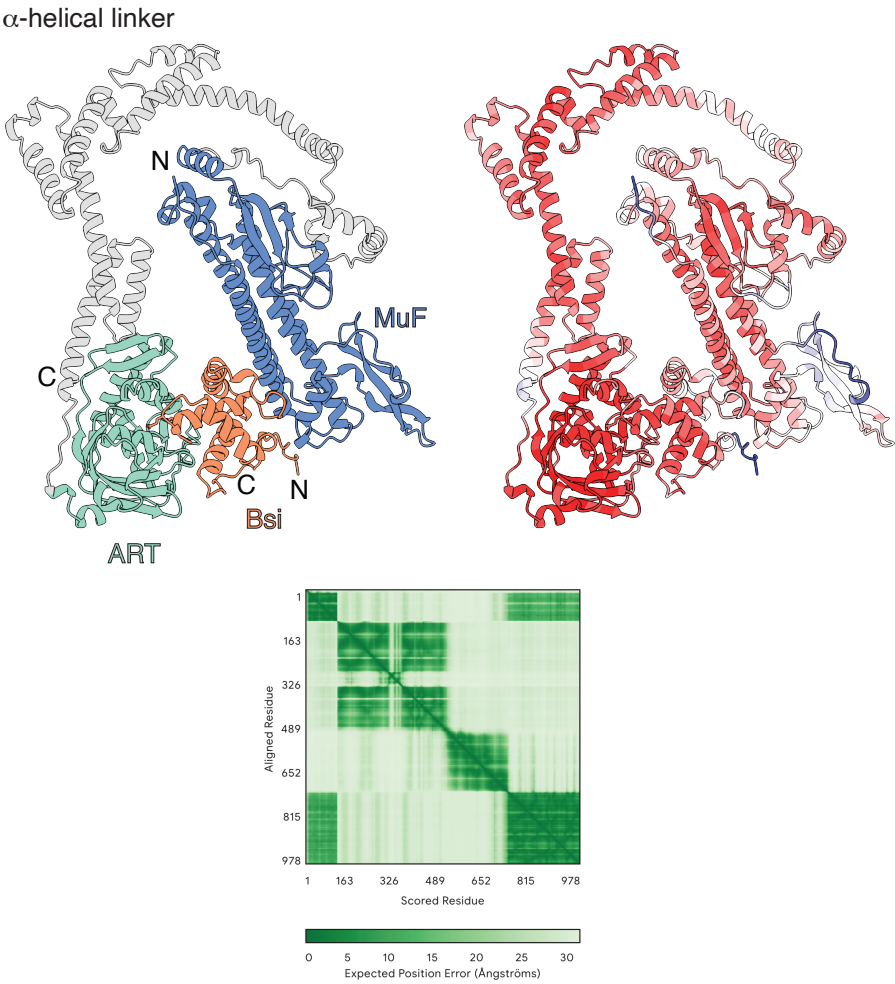

Supplement: Figure S7 [file mmc7.pdf]

Figure S8

A

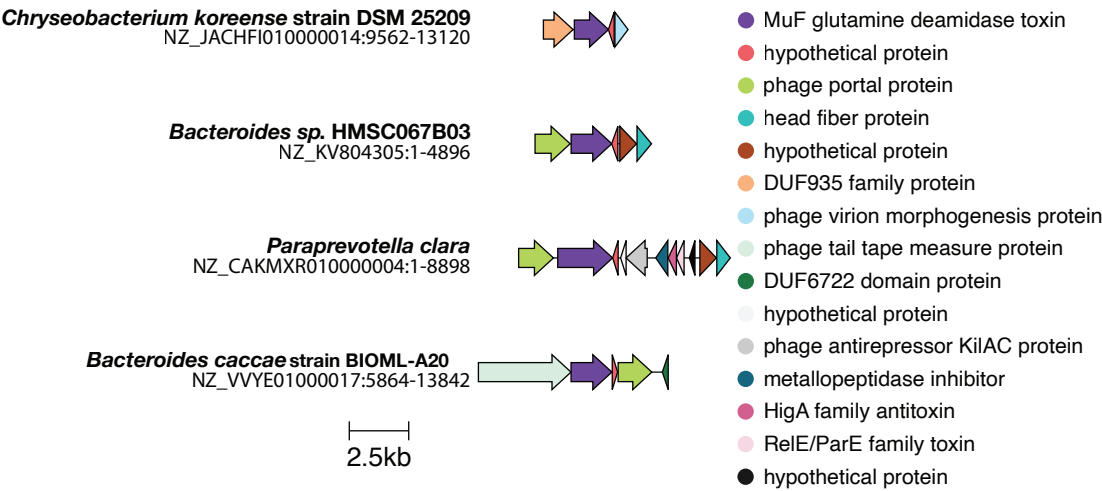

B

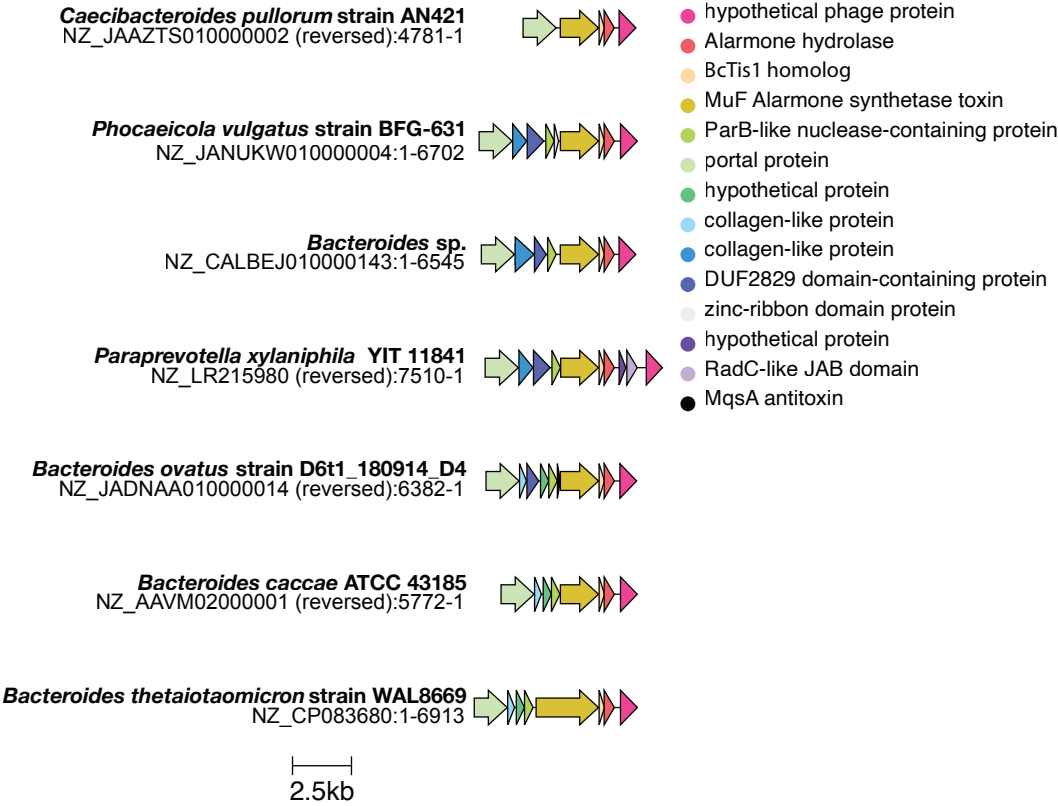

Supplement: Figure S8 [file mmc8.pdf]

Figure S9

A

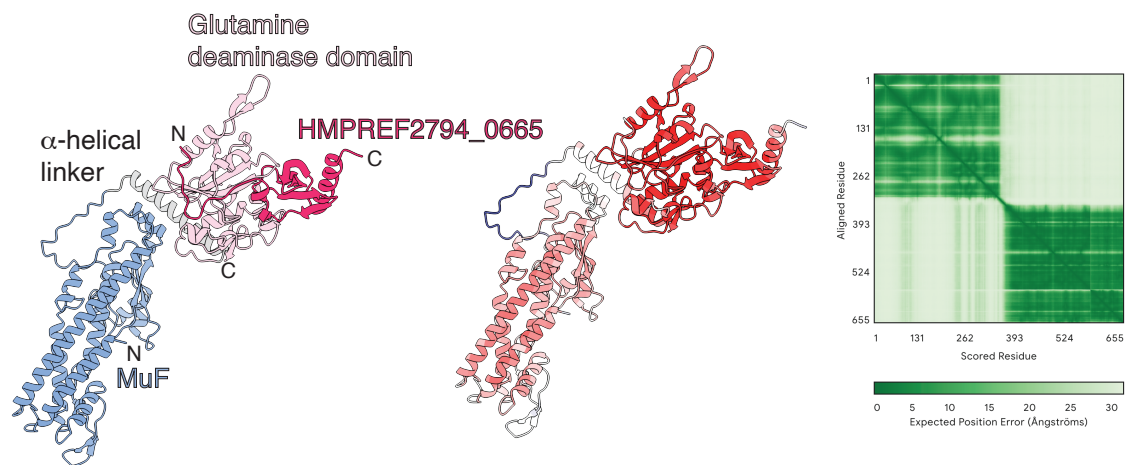

B

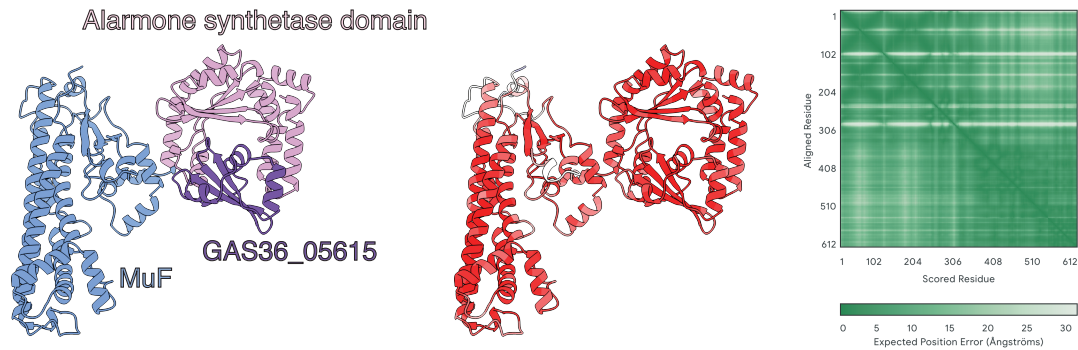

Supplement: Figure S9 [file mmc9.pdf]
