# Supplementary material for: The antibacterial activity of a prophage-encoded fitness factor is neutralized by two cognate immunity proteins
Source: J Biol Chem. 2024 Nov 16;300(12):108007. doi: 10.1016/j.jbc.2024.108007 (PMC11699363; doi:10.1016/j.jbc.2024.108007)
Supplement: Supporting information [file mmc10.docx]

**Figure S1. Triplicate spot plates of Bxa-toxicity and immunity-mediated neutralization.** *A,* Ten-fold serial dilutions of *E. coli* expressing either empty vector (Ctrl), full length Bxa or the catalytic mutant (Bxa*) with or without co-expression of BAH (induced/uninduced) in triplicate. *B,* Ten-fold serial dilutions of *E. coli* expressing either empty vector (Ctrl), the N-terminal and middle domains of Bxa (Bxa_NT-MD_; Bxa_1-621_) or the C-terminal toxin domain (Bxa_CT_; Bxa_622-869_) with or without co-expression of BAH (induced/uninduced) in triplicate. *C,* Ten-fold serial dilutions of *E. coli* encoding plasmid-borne Bxa_CT_ BAH, and BAH^E15A^ plated on media for the induction of either BAH (top) or BAH^E15A^ (bottom) in triplicate. *D*, Ten-fold serial dilutions of *E. coli* containing Bxa_CT_, BAH and either empty vector (Ctrl), or vector encoding BACSTE_RS09440 (RS09440) or BACSTE_RS09450 (RS09450) plated on solid media with inducer for either BAH or the putative immunities in triplicate. *E*, Ten-fold serial dilutions of *E. coli* containing Bxa_CT_, BAH and either empty vector (Ctrl) or vector encoding wild-type Bsi (WT), Bsi^K89E^ (K89E), Bsi^Y90N^ (Y90N), or Bsi^E92K^ (E92K) on plated solid media with or without Bsi induction done in triplicate.

**Figure S2. The ADP-ribosylation profiles of Bxa* and Bxa_CT_* are equally diminished.** Anti-ADPr immunoblot against *E. coli* cells expressing empty vector (Ctrl), Bxa* or Bxa_CT_*. Non-specific bands from the PAGE of these samples stained with Coomassie Brilliant Blue are used as a loading control (LC).

**Figure S3. Modification of Hcp2 reveals residue targeted by Bxa during intoxication.** *A*, Anti-ADPr immunoblot against Hcp2 purified from lysate of *E. coli* expressing empty vector (left) or from lysate of *E. coli* cells containing Bxa_CT_ and BAH expressing Bxa_CT_ (right). *B*, Intact-MS on modified-Hcp2 (Hcp2-ADPr). *C*, Selected spectra from the tandem mass spectrometry of m/z = 905.42 [M+3H].

**Figure S4. Bxa has a comparatively lower ADP-ribosylation profile to Tre1_Tox_.** Anti-ADPr immunoblot of *E. coli* cells collected after expression of empty vector (Ctrl), the ADP-ribosyltransferase toxin domain of Tre1 (Tre1_Tox_), previously defined by Ting et al. (2018), or Bxa for 30 minutes.

**Figure S5. BAH is predicted to be an α–helical globular protein.** Structural prediction of BAH returns an α–helical globular protein (in yellow; left). Predicted local distances difference tests (pLDDT) per position are mapped onto the multimer model (right); higher pLDDT (red) indicates a more confidence prediction.

**Figure S6. Bsi and BACSTE_RS09440 are predicted to be α-helical proteins.** *A*, Structural prediction of BACSTE_RS09440 with AlphaFold3 returns a helix-turn-helix containing protein (left). Predicted local distances difference tests (pLDDT) per position are mapped onto the model (right); higher pLDDT (red) indicates higher confidence. *B*, Structural prediction of Bsi with Alphafold3 returns a globular α-helical protein (left). Predicted local distances difference tests (pLDDT) per position are mapped onto the model (right); higher pLDDT (red) indicates higher confidence.

**Figure S7.** **Alphafold3 positions Bsi in the active site of Bxa.** AlphaFold3 generated structural prediction of Bxa and Bsi interaction interface (left). Bsi (orange) is positioned within the active site of the ART domain of Bxa (green). Predicted local distances difference tests (pLDDT) per position are mapped onto the multimer model (right); higher pLDDT (red) indicates a more confidence prediction. Predicted alignment error in A of all residues against all residues for the top ranked model (bottom). Low error corresponds (darker green) corresponds to well-defined relative domain positions.

**Figure S8. webFlags analysis of ORFs reveal co-occurrence with both Apk2-like and glutamine deaminase MuF proteins.** *A*, Gene diagram of HMPREF2794_0665 and its homologs, which co-occur with MuF proteins possessing a glutamine deaminase C-terminal extension. *B*, Gene diagram of GAS36_05615 and its homologs, which are found to co-occur both with alarmone synthetase containing MuF proteins and alarmone hydrolases.

**Figure S9. Alphafold3 modelling predicts protein-protein interactions with the MuF and their co–occurring small ORFs.** *A*, Structural prediction of the glutamine deaminase domain and HMPREF2794_0665 interaction interface (left). HMPREF2794_0665 (dark pink) is positioned within the predicted active site of the toxin domain (light pink). Predicted local distances difference tests (pLDDT) per position are mapped onto the multimer model (middle); higher pLDDT (red) indicates a more confidence prediction. Predicted alignment error in A of all residues against all residues for the top ranked model (left). Low error corresponds (darker green) corresponds to well-defined relative domain positions. *B*, Structural prediction of the alarmone synthetase domain and GAS36_05615 interaction interface (left). GAS36_05615 (dark purple) is positioned within the predicted active site of the toxin domain (light purple). Predicted local distances difference tests (pLDDT) per position are mapped onto the multimer model (middle); higher pLDDT(red) indicates a more confidence prediction. Predicted alignment error in A of all residues against all residues for the top ranked model (left). Low error corresponds (darker green) corresponds to well-defined relative domain positions.

Table S1: PDBePISA Bsi-Bxa Scores (Hydrogen Bonds)

| Structure #1 (Bxa) | Distance (Å) | Structure #2 (Bsi) |
| --- | --- | --- |
| A: SER 768 [N] | 2.78 | B: ASP 28 [OD1] |
| A: ASN 765 [ND2] | 2.72 | B: GLN 30 [OE1] |
| A: ARG 691 [NH1] | 2.86 | B: GLU 60 [OE1] |
| A: ARG 719 [NH2] | 3.20 | B: TYR 90 [O] |
| A: ARG 679 [NH1] | 3.64 | B: GLU 92 [O] |
| A: THR 675 [0G1] | 3.01 | B: GLU 92 [O] |
| A: SER 757 [N] | 2.88 | B: GLU 92 [OE1] |
| A: TYR 667 [OH] | 3.61 | B: GLU 92 [OE1] |
| A: ARG 679 [NH2] | 2.39 | B: GLU 92 [OE2] |
| A: SER 672 [OG] | 3.19 | B: THR 94 [OG1] |
| A: ALA 791 [O] | 2.95 | B: TRP 38 []NE1 |
| A: TYR 801 [O] | 3.79 | B: ARG 53 [NH2] |
| A: ALA 669 [O] | 2.90 | B: ASN 85 [ND2] |
| A: GLU 818 [OE1] | 2.69 | B: ARG 86 [NH1] |
| A: THR 668 [O] | 3.22 | B: ARG 86 [NH1] |
| A: TYR 667 [O] | 2.32 | B: LYS 89 [NZ] |
| A: GLU 676 [OE1] | 2.75 | B: THR 94 [N] |

Table S2: PDBePISA Bsi-Bxa Scores (Salt Bridges)

| Structure #1 (Bxa) | Distance (Å) | Structure #2 (Bsi) |
| --- | --- | --- |
| A: ARG 691 [NH1] | 2.86 | B:GLU 60 [OE1] |
| A: ARG 719 [NH2] | 3.69 | B: GLU 92 [OE1] |
| A: ARG 719 [NH1] | 3.90 | B: GLU 92 [OE1] |
| A: ARG 679 [NH1] | 3.10 | B: GLU 92 [OE2] |
| A: ARG 679 [NH2] | 2.39 | B: GLU 92 [OE2] |
| A: ARG 719 [NH2] | 3.10 | B: GLU 92 [OE2] |
| A: GLU 818 [OE1] | 2.69 | B: ARG 86 [NH1] |
| A: GLU 820 [OE1] | 3.81 | B: LYS 89 [NZA] |

Table S3: Strains used in this study.

| Organism | Genotype | Description | Reference |
| --- | --- | --- | --- |
| Bacteroides stercoris ATCC 43185 |  | θBxa lysogen | [15] |
| *E. coli* XL-1 Blue | *recA1* *endA1* *gyrA96 thi-1 hsdR17 supE44 relA1 lac* [F´ *proAB lacI*^q^ Z∆*M15* Tn*10* (Tet^R^)] | Cloning strain | Agilent |
| *E. coli* BL21 (DE3) CodonPlus | F^-^ ompT gal dcm lon hsdS_B_(r_B_^-^ m_B_^-^) λ(DE3) pLysS(cm^R^) | Protein expression strain | Novagen |
| *E. coli* BL21 (DE3) pLysS | F– ompT gal dcm lon hsdSB(rB–mB–) λ(DE3 [lacI lacUV5-T7p07 ind1 sam7 nin5]) [malB+]K-12(λS) pLysS[T7p20 orip15A](cm^R^) | Protein expression strain | Novagen |

Table S4: Plasmids used in this study.

| Plasmid | Relevant Features | Reference |
| --- | --- | --- |
| pSCrhaB2-CV | Expression vector with *PrhaB*, Tmp^R^ | [23] |
| pPSV39-CV | Expression vector with *lacI*, *lacUV5* promoter, Gm^R^ | [59] |
| pETDuet-1 | Co-expression vector with *lacI*, T7 promoter, N-terminal His_6_ tag | Novagen |
| pET29B | Expression vector with *lacI*, T7 promoter, C-terminal His_6_ tag | Novagen |
| pBAD33 | Expression vector with *Pbad*, Cm^R^ | [60] |
| pSCrhaB2-CV::Bxa | Expression vector for Bxa | This study |
| pSCrhaB2-CV::Bxa_1-621_ | Expression vector for Bxa_NT-MD_ (residues 1-621) | This study |
| pSCrhaB2-CV::Bxa_622-CT_ | Expression vector for Bxa_CT_ (residues 622-869) | This study |
| pSCrhaB2-CV::Bxa^E820A^ | Expression vector for Bxa* (E820A) | This study |
| pSCrhaB2-CV::Bxa_CT_^E820A^ | Expression vector for Bxa_CT_* (residues 622-869, E820A) | This study. |
| pPSV39-CV::BAH | Expression vector for BAH | This study |
| pBAD33::BAH^E15A^ | Expression vector for BAH^E15A^ | This study. |
| pBAD33::Bsi | Expression vector for Bsi | This study |
| pBAD33::Bsi^K89E^ | Expression vector for Bsi^K89E^ | This study |
| pBAD33:: Bsi^Y90N^ | Expression vector for Bsi^Y90N^ | This study |
| pBAD33::Bsi^E92K^ | Expression vector for Bsi^E92K^ | This study |
| pBAD33::BACSTE_RS09440 | Expression vector for BACSTE_RS09440 | This study |
| pETDuet-1::Bxa*::Bsi | Co-expression vector for x6His-Bxa* and Bsi | This study |
| pETDuet-1::Bxa*::BAH | Co-expression vector for x6His-Bxa* and Bsi | This study |
| pETDuet-1::Empty::Bsi | Expression vector for Bsi | This study |
| pETDuet-1::Empty::BAH | Expression vector for BAH | This study |
| pSCrhaB2-CV::Tre1_Tox_ | Expression vector for Tre1_Tox_ | [28] |
| pPSV39-CV::Tri1 | Expression vector for Tri1_Tox_ | [28] |
| pET29::Hcp2 | Expression vector for Hcp2 | [28] |
| pET29::BAH | Expression vector for BAH | This study |
| pET29::BAH^E15A^ | Expression vector for BAH^E15A^ | This study |
